# Supplementary material for: Novel Driver Strength Index highlights important cancer genes in TCGA PanCanAtlas patients
Source: PeerJ. 2022 Aug 11;10:e13860. doi: 10.7717/peerj.13860 (PMC9375969; doi:10.7717/peerj.13860)
Supplement: Supplemental Information 2 [file peerj-10-13860-s002.zip › COHORTS/genes plots/detailed/2021_8_16_14_9_distribution_events_detailed_females_33.pdf]

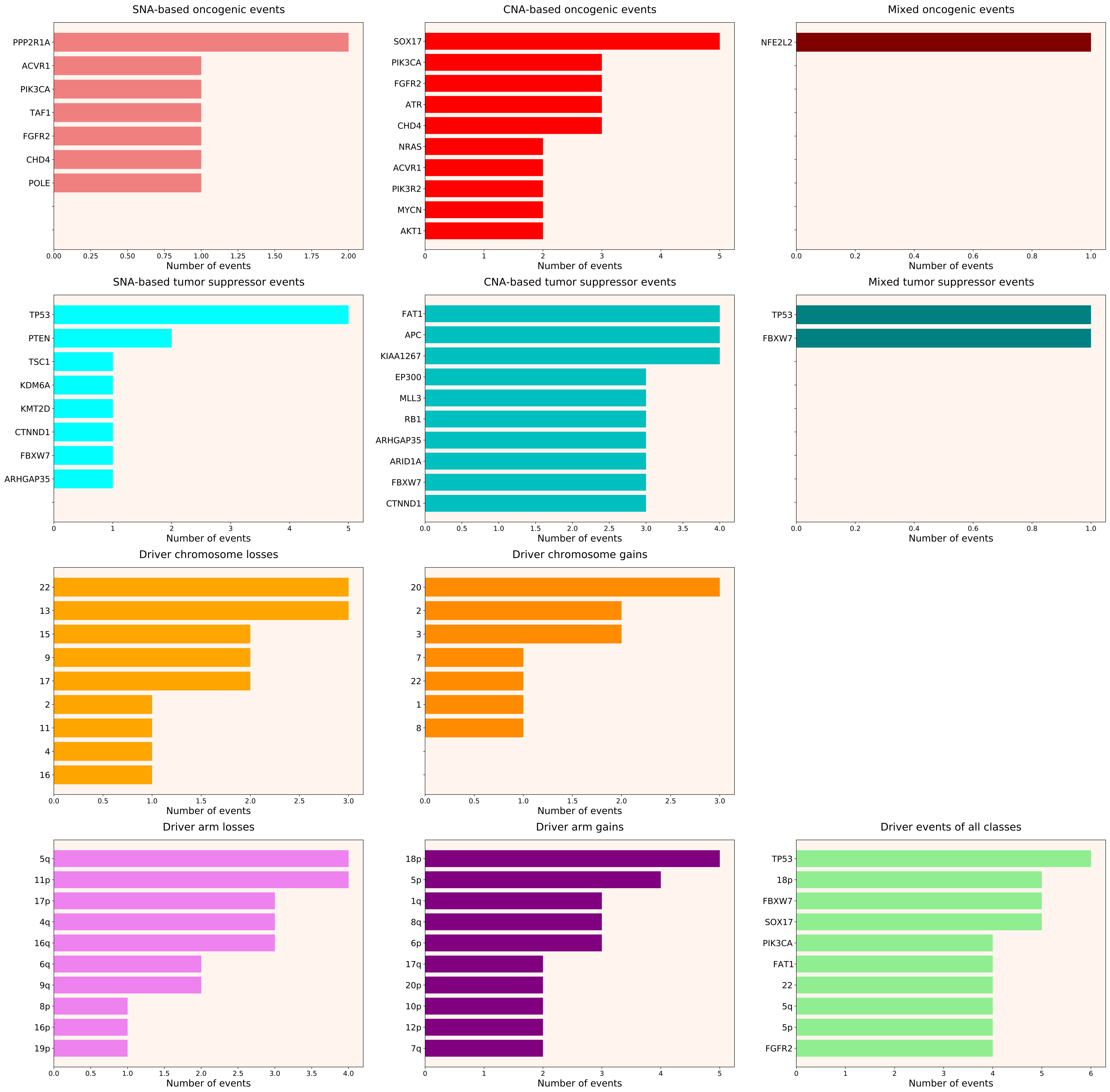
SNA-based oncogenic events
PPP2R1A
ACVR1
PIK3CA
TAF1
FGFR2
CHD4
POLE
0.00 0.25 0.50 0.75 1.00 1.25 1.50 1.75 2.00
Number of events
CNA-based oncogenic events
SOX17
PIK3CA
FGFR2
ATR
CHD4
NRAS
ACVR1
PIK3R2
MYCN
AKT1
0 1 2 3 4 5
Number of events
Mixed oncogenic events
NFE2L2
0.0 0.2 0.4 0.6 0.8 1.0
Number of events
SNA-based tumor suppressor events
TP53
PTEN
TSC1
KDM6A
KMT2D
CTNND1
FBXW7
ARHGAP35
0 1 2 3 4 5
Number of events
CNA-based tumor suppressor events
FAT1
APC
KIAA1267
EP300
MLL3
RB1
ARHGAP35
ARID1A
FBXW7
CTNND1
0.0 0.5 1.0 1.5 2.0 2.5 3.0 3.5 4.0
Number of events
Mixed tumor suppressor events
TP53
FBXW7
0.0 0.2 0.4 0.6 0.8 1.0
Number of events
Driver chromosome losses
22
13
15
9
17
2
11
4
16
0.0 0.5 1.0 1.5 2.0 2.5 3.0
Number of events
Driver chromosome gains
20
2
3
7
22
1
8
0.0 0.5 1.0 1.5 2.0 2.5 3.0
Number of events
Driver arm losses
5q
11p
17p
4q
16q
6q
9q
8p
16p
19p
0.0 0.5 1.0 1.5 2.0 2.5 3.0 3.5 4.0
Number of events
Driver arm gains
18p
5p
1q
8q
6p
17q
20p
10p
12p
7q
0 1 2 3 4 5
Number of events
Driver events of all classes
TP53
18p
FBXW7
SOX17
PIK3CA
FAT1
22
5q
5p
FGFR2
0 1 2 3 4 5 6
Number of events
